# Association of Chronic Inflammation-Associated Cancer With Cytokines

**DOI:** 10.7759/cureus.92239

**Published:** 2025-09-13

**Authors:** Hiroshi Nakase, Yuta Shimomori

**Affiliations:** 1 Division of Gastroenterology and Hepatology, Department of Internal Medicine, Sapporo Medical University School of Medicine, Sapporo, JPN

**Keywords:** cancer therapy and biology, cytokine, regulatory t cells (tregs), tumor associated macrophages, tumor microenvironment

## Abstract

Chronic inflammation supports tissue repair while inadvertently promoting carcinogenesis. Cytokines - critical immune system-signaling molecules - mediate the inflammatory response and establish a cancer-conducive microenvironment. Proinflammatory cytokines, such as tumor necrosis factor-α, interleukin-6, and interleukin-1β, are frequently overexpressed in chronic inflammation and facilitate tumor progression through cell proliferation, survival, angiogenesis, and metastasis. Anti-inflammatory cytokines (e.g., interleukin-10) suppress immune surveillance, which predisposes to tumor growth. Chronic inflammation-induced dysregulation of cytokine networks induces sustained transcriptional factor activation (e.g., nuclear factor-kappa B and signal transducer and activator of transcription 3) that orchestrate gene expression for cell survival and immune evasion. Cytokines influence immune cell recruitment and polarization for immunosuppression and tumor promotion. Emerging evidence highlights the role of cytokines in epithelial-to-mesenchymal transition, a key process in metastasis. Targeting the cytokine-signaling pathway constitutes a promising cancer treatment option. Monoclonal antibodies and small-molecule cytokine inhibitors and their receptors could mitigate tumor-promoting inflammation. Understanding the complex interplay among chronic inflammation, cytokines, and cancer progression is critical for identifying biomarkers and developing targeted therapies to improve clinical outcomes. This review underscores the significance of cytokines in the chronic inflammation-cancer linkage and as potential therapeutic targets. Further research is essential to elucidate intricate cytokine mechanisms in the inflammatory tumor microenvironment for clinical interventions.

## Introduction and background

Chronic inflammation is a well-established risk factor for various cancers, including malignancies of the stomach, liver, colon, and pancreas. The sustained activation of the immune system creates a pro-tumorigenic environment that is driven by the interplay of immune cells, cytokines, and other mediators. Cytokines, small signaling proteins released by immune and non-immune cells, play a central role in regulating inflammation and the immune response. Cytokines exert diverse and sometimes opposing effects on cancer biology, reflecting their classification into distinct functional groups. Pro-inflammatory cytokines, such as tumor necrosis factor-alpha (TNF-α), interleukin (IL)-1β, and IL-6, promote tumor progression by enhancing angiogenesis, promoting cell survival, and creating immunosuppressive microenvironments.

In contrast, type I interferons and interferon-gamma (IFN-γ) are key anti-tumor cytokines that stimulate antigen presentation, activate cytotoxic T lymphocytes, and suppress tumor growth. Growth factors, including transforming growth factor-β (TGF-β) and vascular endothelial growth factor (VEGF), support cancer cell proliferation, invasion, and metastasis; however, TGF-β can also function as a tumor suppressor in the early stages. Chemokines regulate leukocyte trafficking, thereby shaping immune infiltration into tumors, with some subsets recruiting effector cells while others attract regulatory populations that facilitate immune evasion. Immunoregulatory cytokines, such as IL-10, dampen anti-tumor immunity, thereby allowing tumor persistence. Thus, cytokines act as critical modulators of tumor-immune interactions, and therapeutic strategies increasingly aim to block tumor-promoting cytokines or harness anti-tumor cytokines to enhance immune-mediated cancer control. Moreover, chronic exposure to specific cytokines can promote cancer development through mechanisms such as DNA damage, inhibition of apoptosis, promotion of angiogenesis, and immune evasion. This review aimed to explore the association between chronic inflammation-associated cancers and cytokines, with a focus on the molecular mechanisms through which cytokines contribute to tumorigenesis, tumor progression, and metastasis.

## Review

Chronic inflammation and cancer: an overview 

Rudolf Virchow (1821-1902), considered the "Father of Modern Pathology," made seminal contributions that have significantly shaped our understanding of disease at the cellular level. In the 19th century, Virchow first reported the presence of white blood cells (immune cells) that infiltrate cancerous tissues. This finding led him to propose a groundbreaking hypothesis: chronic inflammation may contribute to the development of tumors [1]. Virchow postulated that persistent inflammation fosters a microenvironment that is rich in cytokines, growth factors, and reactive oxygen species (ROS), all of which can induce genetic mutations, promote cell proliferation, and impair standard apoptotic mechanisms [2]. These factors collectively create conditions that are favorable for neoplastic transformation and tumor progression [3,4]. Furthermore, Virchow emphasized that chronic infections and unresolved inflammation can drive continuous tissue remodeling, thereby enhancing the risk of carcinogenesis [2]. Modern studies have validated Virchow’s hypothesis and clearly demonstrated associations between chronic inflammatory conditions, such as inflammatory bowel disease, chronic viral hepatitis, and Helicobacter pylori-induced gastritis, and increased cancer risk [4-6]. Virchow's work established the crucial link between pathology, immunology, and oncology, and his concept of "inflammation-driven cancer" remains a cornerstone in current tumor biology research [1,3,6].

**Figure 1:**
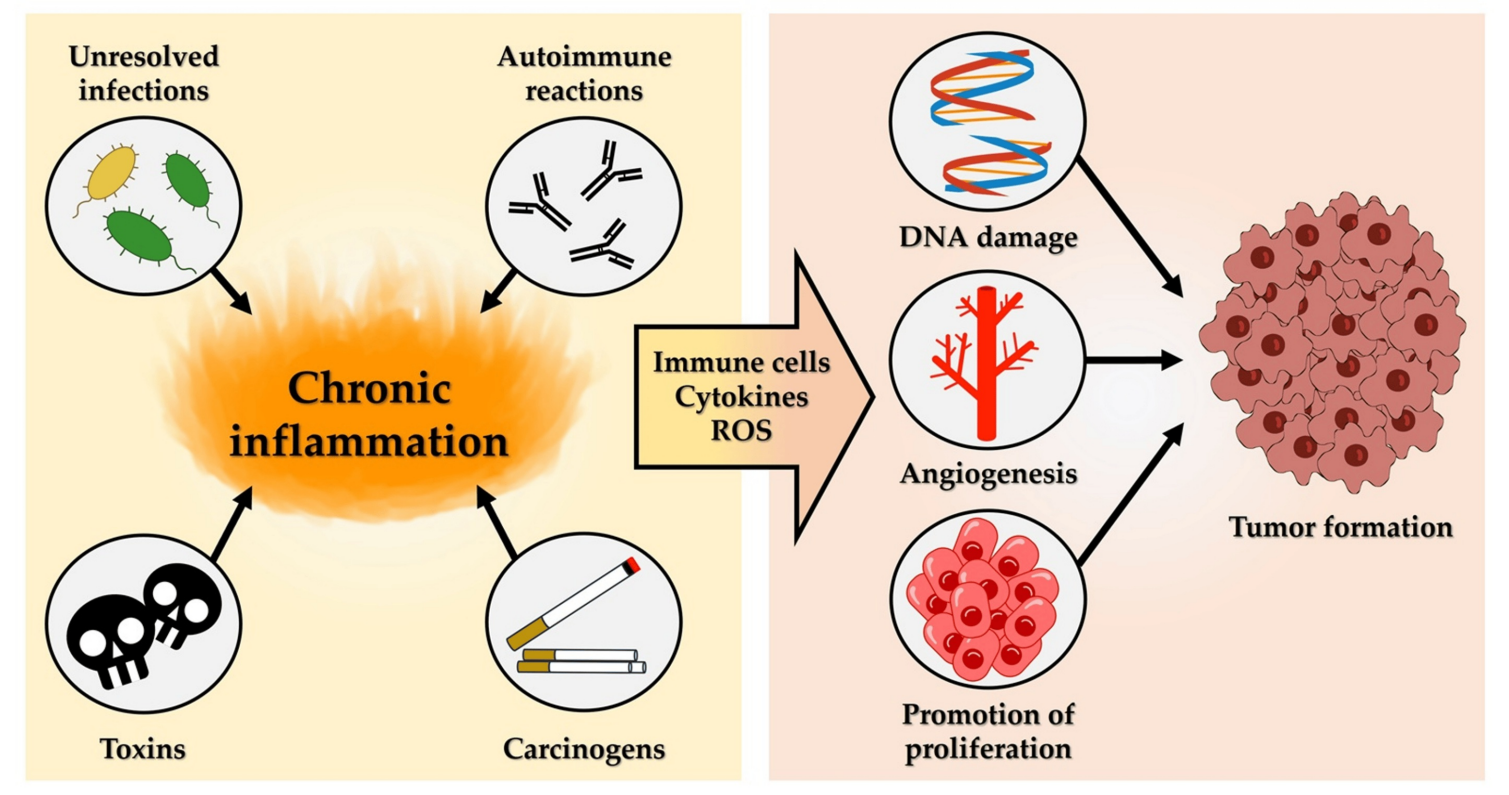
Chronic inflammation and cancer: An overview. Unresolved infections, autoimmune reactions, or long-term exposure to irritants, such as toxins and carcinogens, can cause chronic inflammation, which leads to immune cell infiltration, proinflammatory cytokine release and ROS production. Sustained chronic inflammation induces DNA damage and angiogenesis, and promotes proliferation, all of which leads to carcinogenesis. Abbreviations: DNA, deoxyribonucleic acid; ROS, reactive oxygen species

Chronic inflammation is a hallmark of cancer, which acts as a double-edged sword [7]. Although acute inflammation typically eliminates pathogens and promotes tissue repair, chronic inflammation creates an environment that is conducive to the development of carcinogenesis. Persistent inflammatory conditions, such as inflammatory bowel disease, chronic hepatitis, and chronic gastritis, are associated with a higher risk of cancers in organs, such as the colon, liver, and stomach, respectively [8-10]. During chronic inflammation, immune cells infiltrate tissues and release a wide range of proinflammatory cytokines, which can lead to genetic mutations, promote cell proliferation, and inhibit normal apoptotic processes. This sustained immune response is frequently a result of unresolved infections, autoimmune reactions, or prolonged exposure to irritants, such as toxins or carcinogens [2,4,11] (Figure 1). 

The Key Pro-carcinogenic Mechanisms of Chronic Inflammation

DNA damage: ROS and nitrogen species produced by inflammatory cells can cause DNA damage and lead to mutations [5,12,13].

Promotion of proliferation: Cytokines such as various interleukins (IL-2, IL-3, IL-4, IL-5, IL-6, IL-7, and IL-15), as well as TNF-α, colony-stimulating factors, stimulate cell proliferation, which allows mutated cells to expand and accumulate further genetic alterations [3,5,14].

Inhibition of apoptosis: Anti-apoptotic cytokines (IL-3 and stem cell factor, erythropoietin and anamorsin) prevent or inhibit programmed cell death (apoptosis), thereby promoting cell survival and health, particularly in response to injury or stress [4,15].

Angiogenesis: Chronic inflammation promotes neovascularization to sustain tumor growth [16].

Immune evasion: Tumors exploit cytokines to create an immunosuppressive environment, which helps them avoid immune detection [17,18].

The role of cytokines in inflammation-associated cancer

**Figure 2:**
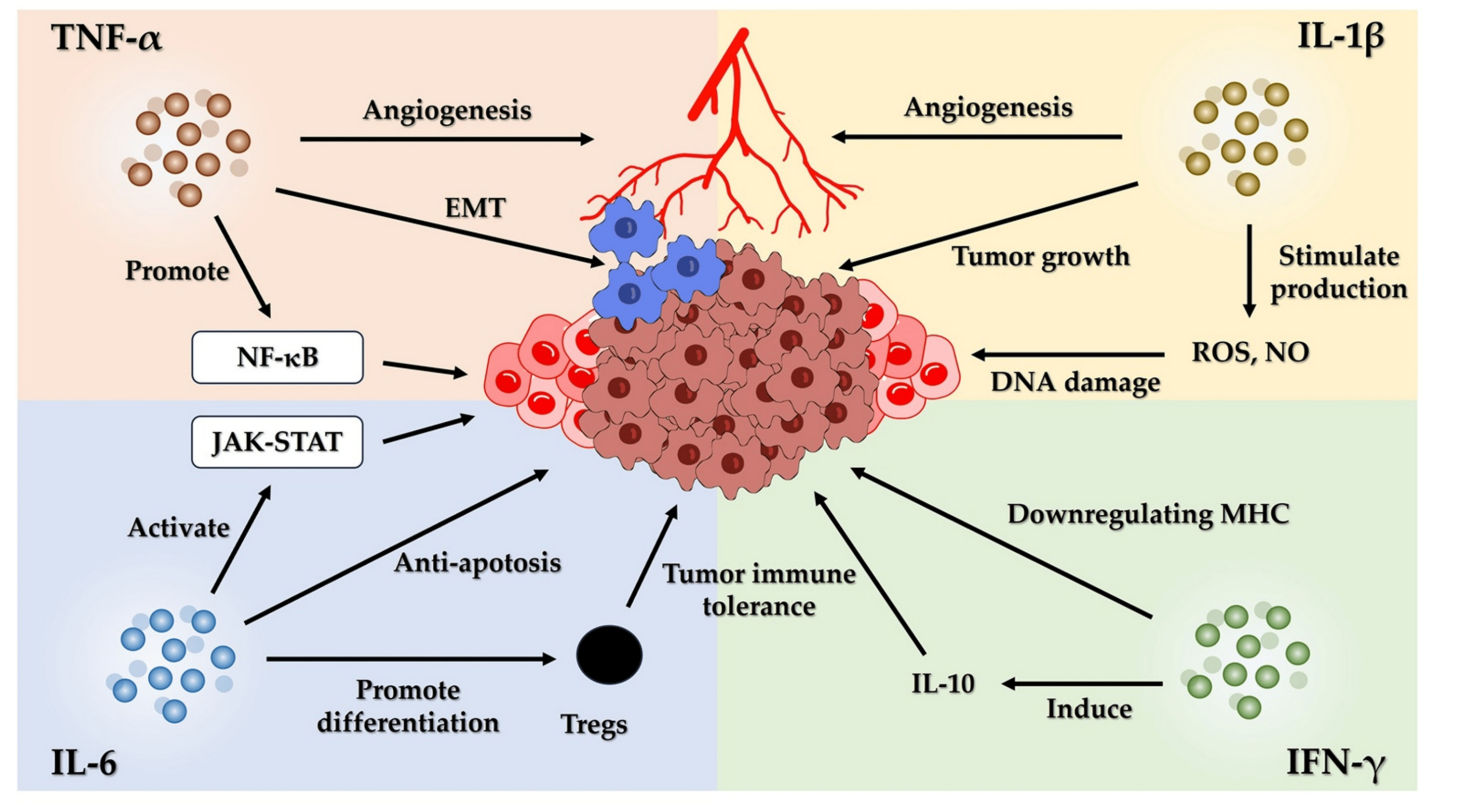
The role of cytokines in inflammation-associated cancer. Cytokines play a central role in the relationship between chronic inflammation and cancer. TNF-α, IL-6, IL-1β, and IFN-γ create an environment that promotes cancer cell survival, proliferation, and immune evasion. Abbreviations: DNA, deoxyribonucleic acid; EMT, epithelial–mesenchymal transition; IFN-γ, interferon-gamma; IL, interleukin; JAK, Janus kinase; MHC, major histocompatibility complex; NF-κB, nuclear factor-kappa B; NO, nitric oxide; ROS, reactive oxygen species; STAT, signal transducer and activator of transcription; TNF-α, tumor necrosis factor-alpha; Treg, regulatory T

Cytokines play a central role in the relationship between chronic inflammation and cancer as they mediate various immune responses and modulate the tumor microenvironment (TME). Specific proinflammatory cytokines, such as TNF-α, IL-6, IL-1β, and IFN-γ, play critical roles in promoting tumorigenesis by creating an environment that favors cancer cell survival, proliferation, and immune evasion (Figure 2).

To clearly understand the experimental data from mouse models, it is important to recognize the differences in the functions of cytokines in humans and in rodents. Studies using comparative genomic and functional approaches have highlighted species-specific cytokine biology that emphasize the limitations of rodent models in predicting human cytokine responses. Although core cytokine pathways, such as IL-6 and TNF-α, are conserved, there exist differences between human and rodent interferon and inflammasome responses [19,20].

TNF-α

Bruce Beutler's pioneering research in the 1980s elucidated the role of TNF as a central mediator of inflammation and septic shock. Beutler discovered that excessive TNF production triggers systemic inflammatory responses, which lead to lethal shock syndromes [21]. Soluble TNF receptors, known as "receptor bodies," which bind circulating TNF and neutralize its activity, offer a therapeutic strategy to mitigate TNF-driven pathologies [22]. Walter Fiers significantly advanced the field by being the first to clone and sequence human TNF-α, establish its molecular structure, and confirm its cytotoxic and proinflammatory functions [23]. Fiers' research constitutes the foundational work for understanding TNF as both a physiological regulator and a pathological agent in diseases, such as cancer and autoimmune conditions.

In response to inflammation, TNF-α is produced by macrophages, lymphocytes, and other cells that play a vital role in immune defense against pathogens; however, chronic TNF-α exposure has been implicated in tumorigenesis [24]. Elevated TNF-α levels have been associated with several cancers, including colorectal cancer, gastric cancer, hepatocellular carcinoma (HCC), and pancreatic cancer [2]. TNF-α binds to TNF receptor 1 (TNFR1) or TNFR2, triggering recruitment of adaptor proteins such as TRADD and TRAF2. This activates downstream nuclear factor-kappa B (NF-κB) and MAPK signaling cascades. NF-κB translocates into the nucleus, inducing transcription of genes that promote cell survival and proliferation, including cyclins and anti-apoptotic factors (e.g., Bcl-XL, c-IAPs). Simultaneously, MAPK/ERK signaling enhances expression of growth-related genes. Although TNF-α can also induce apoptosis via caspase-8 activation, in many contexts the NF-κB-mediated prosurvival signals dominate, enabling cell proliferation and contributing to chronic inflammation and tumor progression.

The Mechanisms Through Which TNF-α Promotes Cancer

Promotion of NF-κB signaling: TNF-α activates the NF-κB pathway, which regulates genes that are involved in cell survival, proliferation, and inflammation. Chronic activation of NF-κB promotes the survival of cells with damaged DNA and enhances resistance to apoptosis [25].

Angiogenesis: TNF-α induces the production of VEGF, which promotes neovascularization to support tumor growth [26].

Epithelial-mesenchymal transition (EMT): TNF-α can induce EMT - a process whereby epithelial cells gain mesenchymal characteristics, which enhances their migratory and invasive abilities and thus promotes metastasis [27].

The Association of TNFα With the Pathophysiology of Several Cancers

Breast cancer: TNF-α is frequently elevated in breast cancer tissues and is associated with aggressive phenotypes and poor prognosis. It promotes tumor cell proliferation and invasiveness by inducing EMT and upregulating matrix metalloproteinases (MMPs) [28]. Furthermore, TNF-α enhances the expression of pro-inflammatory and pro-angiogenic factors such as IL-6 and VEGF, contributing to a tumor-supportive microenvironment [29].

Colorectal cancer (CRC): In CRC, chronic inflammation driven by TNF-α contributes to tumor initiation and progression. TNF-α induces DNA damage and promotes a pro-inflammatory microenvironment conducive to malignant transformation [30]. The cytokine also facilitates the recruitment and activation of myeloid-derived suppressor cells (MDSCs), which suppress anti-tumor immune responses [31]. In mouse models of colitis-associated colorectal cancer, TNF-α promoted tumor growth through its interaction with the NF-κB pathway, which suggests that targeting TNF-α constitutes a potential therapeutic strategy in inflammation-associated cancers [32].

Pancreatic cancer: Pancreatic ductal adenocarcinoma (PDAC) is characterized by a dense stromal reaction and persistent inflammation. TNFα expression is elevated in PDAC and correlates with disease progression and resistance to apoptosis. It promotes tumor-stroma interactions and desmoplasia, enhancing tumor growth and immune evasion [33]. Targeting TNF-α in preclinical models has been shown to reduce tumor burden and improve response to chemotherapy [34].

Dual role in cancer: While TNF-α has tumor-promoting effects, it was initially named for its ability to cause necrosis of tumors in animal models. At high concentrations, TNF-α can induce apoptosis and disrupt tumor vasculature [35]. This has led to the development of localized TNF-α-based therapies, such as isolated limb perfusion for the treatment of sarcoma. However, systemic administration is limited due to severe toxicities.

IL-6

IL-6 has a well-established role in cancer. IL-6 is produced by various immune cells, including macrophages and T cells, and non-immune cells, such as fibroblasts. Chronic IL-6 signaling has been linked to the development of cancers such as colon cancer, lung cancer, and multiple myeloma. IL-6 signals through a receptor complex consisting of the IL-6 receptor α chain (IL-6Rα, either membrane-bound or soluble) and the signal-transducing subunit gp130. Upon IL-6 binding, IL-6Rα associates with gp130, leading to gp130 dimerization and activation of Janus kinases (JAKs) associated with its cytoplasmic domain. Activated JAKs phosphorylate tyrosine residues on gp130, which serve as docking sites for signaling molecules, most notably signal transducer and activator of transcription (STAT)-3. STAT3 becomes phosphorylated, dimerizes, and translocates into the nucleus, where it induces transcription of genes promoting cell cycle progression. In parallel, IL-6-gp130 signaling activates the Ras-MAPK and PI3K-Akt pathways, which further enhance cell survival and proliferation. Dysregulated IL-6 signaling, often through constitutive STAT3 activation, is implicated in tumorigenesis, chronic inflammation, and autoimmune disease. Thus, the IL-6 receptor pathway represents a critical node linking inflammation to cell proliferation and oncogenesis.

The Mechanisms Through Which IL-6 Promotes Cancer

Activation of the JAK/STAT pathway: IL-6 primarily signals through the JAK/STAT pathway, which leads to the transcription of genes involved in cell survival, proliferation, and inflammation [36]. Constitutive activation of the JAK/STAT pathway is commonly observed in various cancers [37].

Anti-apoptotic effects: IL-6 promotes cell survival by upregulating anti-apoptotic proteins, such as Bcl-2 and Bcl-xL, which help damaged cells avoid programmed cell death [38,39].

Immune evasion: IL-6 can create an immunosuppressive environment by promoting the differentiation of regulatory T cells (Tregs) and inhibiting the activity of cytotoxic T cells, which are essential for tumor immune surveillance [40,41].

The Association of IL-6 With the Pathophysiology of Several Cancers

Breast cancer: IL-6 levels are often elevated in the serum and TME of breast cancer patients compared to healthy individuals. High IL-6 levels are associated with larger tumor size, lymph node involvement, and distant metastases. IL-6 contributes to angiogenesis by upregulating VEGF expression. IL-6 enhances EMT, facilitating tumor invasion and metastasis. Elevated IL-6 is linked to endocrine therapy resistance, particularly in estrogen receptor-positive cancers. Triple-negative breast cancers (TNBCs) often show exceptionally high IL-6 levels, associated with poor prognosis. IL-6 fosters an immunosuppressive microenvironment that helps tumors evade immune attack. Targeting IL-6 or its signaling pathways is being investigated as a potential therapeutic strategy in breast cancer [42,43].

CRC: In CRC, IL-6 is increasingly recognized as a critical factor in tumor initiation, progression, and metastasis. Elevated IL-6 levels are associated with poor prognosis in patients with CRC. Serum IL-6 levels are significantly elevated in patients with CRC compared to healthy controls [44]. Higher IL-6 levels are correlated with advanced tumor stage (TNM stage III/IV), presence of lymph node and distant metastases, and poor overall survival [45,46].

Pancreatic cancer: In the serum and TME of patients with pancreatic cancer, IL-6 is significantly elevated [47]. High IL-6 levels correlate with advanced stage, metastasis, and cachexia [48]. IL-6 promotes pancreatic tumor growth via activation of the JAK/STAT3 signaling pathway. It enhances angiogenesis by inducing VEGF production, supporting tumor vascularization. IL-6 facilitates immune evasion by expanding MDSCs and regulatory T cells [49]. Elevated IL-6 is associated with shorter overall survival in pancreatic cancer patients. IL-6 contributes to resistance to chemotherapy in pancreatic cancer models. Blocking IL-6 or its receptor reduces tumor growth and metastasis in preclinical studies. IL-6 may serve as a prognostic biomarker and therapeutic target in pancreatic cancer. Clinical trials are investigating IL-6 pathway inhibitors as part of combination treatments.

HCC: Chronic liver inflammation, frequently owing to hepatitis B/C infection or nonalcoholic steatohepatitis (NASH), leads to sustained IL-6 production by Kupffer cells and hepatocytes. IL-6 activates the JAK/STAT3 signaling pathway, promoting hepatocyte proliferation, angiogenesis, and resistance to apoptosis. Elevated serum IL-6 levels correlate with larger tumor size, vascular invasion, and poor prognosis. This makes IL-6 a potential biomarker and therapeutic target in liver cancer and other inflammation-associated malignancies [50]. IL-6 also fosters an immunosuppressive TME by enhancing regulatory T cell function and inhibiting cytotoxic T cells. Additionally, IL-6 promotes EMT, facilitating metastasis. Genetic polymorphisms in the IL-6 gene are associated with an increased susceptibility to HCC. Targeting the IL-6/STAT3 axis is a promising therapeutic strategy in HCC treatment [51-53].

IL-1β

A potent proinflammatory cytokine, IL-1β is produced by activated macrophages and monocytes in response to infection or tissue injury. Chronic elevation of IL-1β has been associated with several cancers, including lung, breast, and colorectal cancers.

The Mechanisms Through Which IL-1β Promotes Cancer

Inflammation-induced DNA damage: IL-1β stimulates the production of ROS and nitric oxide (NO), which can cause DNA damage and mutagenesis [54].

Promotion of angiogenesis: Similar to TNF-α, IL-1β promotes angiogenesis by inducing VEGF expression [55].

Modulation of the tumor microenvironment: IL-1β can alter the TME by recruiting immune cells, such as neutrophils and macrophages, that support tumor growth and suppress antitumor immune responses [56].

Furthermore, IL-1β promotes metastasis. In pancreatic cancer, IL-1β enhanced the metastatic potential of tumor cells by facilitating their dissemination to distant organs [57].

The Association of IL-1β With the Pathophysiology of Several Cancers

Breast cancer: IL-1β is a proinflammatory cytokine that significantly contributes to the development and progression of breast carcinoma. IL-1β promotes tumor growth by stimulating angiogenesis, tumor cell proliferation, and EMT, facilitating metastasis. It activates key signaling pathways such as NF-κB and MAPK, creating a pro-tumorigenic microenvironment. IL-1β is often elevated in aggressive breast cancer subtypes, particularly TNBC, and is associated with poor prognosis. Tumor-associated macrophages and cancer cells themselves can be sources of IL-1β within the tumor microenvironment. Moreover, IL-1β enhances immune suppression by recruiting MDSCs. Inhibition of IL-1β has shown promise in preclinical models as a potential therapeutic strategy to reduce metastasis and improve immune response [58-60].

CRC: A key proinflammatory cytokine, IL-1β, plays a crucial role in the development and progression of CRC. It promotes tumorigenesis by enhancing chronic inflammation, angiogenesis, and the recruitment of MDSCs, which contribute to immune evasion. IL-1β activates signaling pathways, including NF-κB and the IL-6/STAT3 pathway, leading to increased tumor cell proliferation and survival. Studies have shown that IL-1β is upregulated in both CRC tissues and the tumor microenvironment, particularly in tumor-associated macrophages and stromal cells. Moreover, IL-1β facilitates EMT, a process critical for metastasis. In murine models, IL-1β deficiency or blockade has been shown to reduce tumor growth and metastasis. Elevated IL-1β levels in patient samples are correlated with poor prognosis and advanced disease stage. Therapeutic targeting of IL-1β, including the use of IL-1 receptor antagonists such as anakinra, is being explored in both preclinical and clinical settings for CRC. These findings highlight IL-1β as both a biomarker and a potential therapeutic target in CRC [61-63].

Pancreatic cancer: By promoting inflammation-driven tumorigenesis, IL-1β plays a pivotal role in the development and progression of pancreatic cancer, and contributes to the remodeling of the TME by recruiting immunosuppressive cells, such as tumor-associated macrophages and MDSCs. High IL-1β expression correlates with increased tumor invasiveness and poorer clinical outcomes. Moreover, IL-1β promotes EMT, facilitating metastasis. Pancreatic cancer cells can produce IL-1β or stimulate stromal cells to secrete it, sustaining a chronic inflammatory niche. In preclinical studies, blockade of IL-1β signaling suppressed tumor growth and improved responses to chemotherapy. These findings underscore IL-1β as a potential prognostic marker and therapeutic target in pancreatic ductal adenocarcinoma [57,64,65].

HCC: IL-1β is a key mediator of inflammation and plays a significant role in HCC development. Chronic liver inflammation, often caused by hepatitis virus infection or NASH, leads to IL-1β production by Kupffer cells and hepatic stellate cells. IL-1β promotes hepatocyte proliferation, survival, and angiogenesis. It also facilitates a tumor-promoting microenvironment by recruiting MDSCs and promoting immune evasion. Elevated IL-1β levels are associated with poor prognosis and higher tumor burden in HCC patients. Additionally, IL-1β can enhance EMT, increasing metastatic potential. Preclinical studies show that blocking IL-1β signaling can inhibit HCC progression and enhance anti-tumor immunity, highlighting its potential as a therapeutic target [66-68].

Interferon-Gamma

Unlike other proinflammatory cytokines, IFN-γ is generally regarded as antitumorigenic owing to its role in activating cytotoxic T cells and enhancing immune surveillance. However, under certain conditions, chronic IFN-γ exposure can contribute to cancer progression.

The Mechanisms Through Which IFN-γ May Promote Cancer

Immunoediting: Tumors exposed to prolonged IFN-γ signaling may develop resistance by downregulating major histocompatibility complex (MHC) molecules, making them less visible to cytotoxic T cells [69].

Promotion of immune suppression: IFN-γ can induce the production of other cytokines, such as IL-10, which have immunosuppressive effects and may help tumors evade immune detection [70].

In pancreatic cancer, chronic IFN-γ signaling has been implicated in promoting an immunosuppressive environment that favors tumor growth. However, more research is needed to fully understand the dual roles of IFN-γ in cancer progression and immune surveillance [71].

The Association of IFN-γ With the Pathophysiology of Several Cancers

Breast cancer: In breast cancer, IFN-γ plays a dual role, where it acts both as a tumor suppressor and a modulator of immune escape. As an anti-tumor cytokine, IFN-γ enhances antigen presentation by upregulating MHC class I/II, activates cytotoxic T cells and natural killer (NK) cells, and directly induces apoptosis in tumor cells. High IFN-γ levels are associated with improved prognosis, especially in immunogenic subtypes like TNBC. However, chronic exposure to IFN-γ can induce immune tolerance by upregulating programmed death-ligand 1 (PD-L1) and indoleamine 2,3-dioxygenase (IDO), contributing to immune evasion. Breast cancer cells may also develop resistance to IFN-γ signaling through JAK/STAT pathway alterations. Moreover, IFN-γ plays a role in shaping the TME by influencing stromal and immune cell function. Therapeutic strategies aimed at restoring or modulating IFN-γ signaling are under investigation to enhance immunotherapy responses [72-74].

CRC: In CRC, IFN-γ is a key immunoregulatory cytokine with a significant role. It exerts anti-tumor effects by activating cytotoxic T lymphocytes and NK cells, enhancing tumor antigen presentation through upregulation of MHC class I/II, and inducing apoptosis in CRC cells. IFN-γ also stimulates macrophage activation and promotes Th1 immune responses, which are generally associated with a better prognosis. However, chronic or excessive IFN-γ exposure can upregulate immune checkpoints like PD-L1 and promote IDO expression, contributing to immune escape. In mismatch repair-deficient (dMMR) CRC, IFN-γ signaling is particularly important for immunotherapy response. Loss of IFN-γ pathway components (e.g., JAK1/2 mutations) can predict resistance to immune checkpoint inhibitors. Thus, IFN-γ plays both protective and regulatory roles in CRC progression and treatment [75-77]. 

Pancreatic cancer: In pancreatic cancer, IFN-γ plays a complex role, wherein, depending on the TME, it exhibits both anti-tumor and pro-tumor effects. As an anti-tumor cytokine, IFN-γ can induce tumor cell apoptosis, enhance antigen presentation via MHC class I and II upregulation, and activate cytotoxic T lymphocytes and NK cells. However, chronic or dysregulated IFN-γ signaling can paradoxically promote immune evasion by upregulating immune checkpoints like PD-L1 and contributing to tumor desensitization. In PDAC, IFN-γ expression is often low, but its restoration can enhance the efficacy of immunotherapy. Additionally, IFN-γ may inhibit tumor fibrosis and reduce the dense stromal barrier, potentially improving drug delivery. Thus, modulating IFN-γ activity may offer therapeutic benefit when combined with immune checkpoint inhibitors or chemotherapy [78-80].

HCC: In HCC, IFN-γ plays a complex, context-dependent role. As a key cytokine in anti-tumor immunity, IFN-γ enhances antigen presentation by upregulating MHC class I molecules, activates cytotoxic T lymphocytes and NK cells, and induces apoptosis in HCC cells. Higher intratumoral IFN-γ expression is associated with better prognosis and reduced tumor recurrence. However, chronic IFN-γ exposure may promote immune tolerance by inducing immune checkpoints like PD-L1 and IDO, contributing to tumor immune evasion. Moreover, IFN-γ modulates the TMEt by affecting macrophage polarization and inhibiting angiogenesis. Defective IFN-γ signaling is linked to poor response to immunotherapy in HCC. Thus, while IFN-γ is generally protective, its dysregulation can impair immune surveillance [81-83].

Cytokine networks and the tumor microenvironment

**Figure 3:**
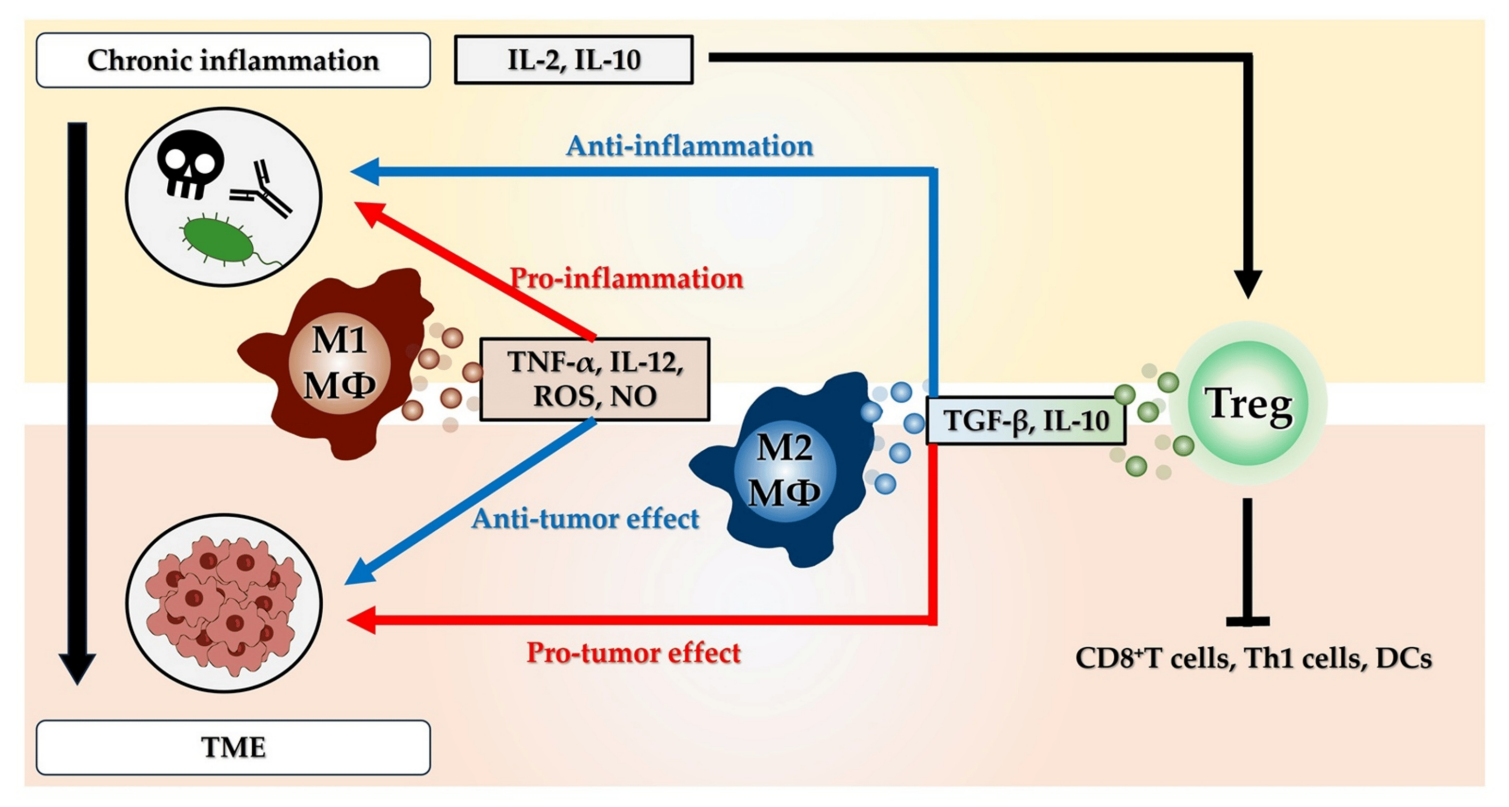
Cytokine networks and the tumor microenvironment. Tumor-associated macrophages are generally classified into M1 and M2 macrophages. M1 macrophages play a role in pathogen clearance and tumor suppression. M2 macrophages and Treg cells suppress not only the inflammation that causes inflammatory carcinogenesis, but also the immune response to cancer. Abbreviations: DC, dendritic cell; IL, interleukin; MΦ, macrophage; NO, nitric oxide; ROS, reactive oxygen species; TGF-β, transforming growth factor-beta; Th, helper T; TME, tumor microenvironment; TNF-α, tumor necrosis factor-alpha; Treg, regulatory T

The TME comprises a dynamic mixture of cancer cells, stromal cells, immune cells, and signaling molecules, including cytokines. The interaction between cancer cells and the immune system is critical for tumor progression, and cytokines are key mediators of this crosstalk. In inflammation-associated cancers, proinflammatory cytokines can create a microenvironment that favors tumor growth, whereas anti-inflammatory cytokines can promote immune evasion and tumor progression (Figure 3).

Tumor-Associated Macrophages

Macrophages are highly plastic immune cells that adapt their phenotype in response to local signals. Tumor-associated macrophages (TAMs) are generally classified into the following categories.

Classically activated (M1) macrophages, which produce proinflammatory cytokines (e.g., IL-12, TNF-α), ROS, and NO, play a crucial role in pathogen clearance and tumor suppression.

Alternatively activated (M2) macrophages, which secrete anti-inflammatory cytokines (e.g., IL-10, TGF-β) and support tissue remodeling, angiogenesis, and tumor progression.

M1-like macrophages promote tissue damage and genomic instability during chronic colitis by releasing inflammatory mediators, such as IL-6, TNF-α, and ROS. These factors can induce mutations and epigenetic changes that contribute to the neoplastic transformation of epithelial cells [4,84-86]. TAMs frequently acquire an M2-like phenotype in the context of cancers, although the M1/M2 dichotomy is an oversimplification. The phenotype of TAMs can vary depending on the stage of inflammation and tumor progression. TAMs produce cytokines, such as IL-10 and TGF-β, which suppress antitumor immune responses and promote tissue remodeling, angiogenesis, and metastasis. Elevated levels of these cytokines are frequently associated with poor prognosis in various cancers, including breast, ovarian, and colorectal cancers [87-89].

Regulatory T Cells

Chronic inflammation is a well-established contributor to carcinogenesis, acting through mechanisms such as DNA damage, cell proliferation, inhibition of apoptosis, and angiogenesis. Tregs play a paradoxical but critical role in modulating immune responses within this inflammatory microenvironment. Although essential for maintaining immune tolerance and preventing autoimmunity, Tregs are increasingly recognized as key facilitators of immune evasion in cancer, particularly those driven by inflammation.

Role of Tregs in the TME

Tregs, typically identified by expression of CD4, CD25, and the transcription factor FOXP3, are often recruited in large numbers to the TME. They suppress the function of effector T cells (especially CD8⁺ cytotoxic T cells and Th1 cells) and dendritic cells, and thereby dampen antitumor immunity. This immunosuppressive function is frequently co-opted by tumors to evade immune surveillance [90].

Recruitment and Expansion of Tregs by Inflammatory Signals

Levels of inflammatory cytokines, such as TGF-β, IL-10, and IL-2, are elevated in chronic inflammation and can facilitate the recruitment, induction, and expansion of Tregs within tissues and tumors. For instance, TGF-β plays a dual role, which involves the promotion of inflammation-induced EMT and stimulation of FOXP3 expression, which convert naïve T cells into inducible Tregs [91].

Tregs in Inflammation-Driven Carcinogenesis

In models of inflammation-driven cancers, such as colitis-associated colorectal cancer, Tregs modulate the local immune balance. Although they may initially limit tissue-damaging inflammation, they ultimately facilitate tumor progression by suppressing antitumor responses and fostering an immune-permissive environment for genetically altered cells to expand [4,92]. Thus, Tregs are central players in the immune landscape of inflammation-driven cancers. Promoting an immunosuppressive microenvironment contributes to tumor initiation, progression, and resistance to treatment. Understanding the dual nature of Tregs in inflammation and immunity is crucial for developing effective and safe cancer immunotherapies.

Cytokine-targeting cancer therapy

Cytokines play pivotal roles in tumor progression, immune evasion, and inflammation-driven carcinogenesis. Consequently, monoclonal antibodies (mAbs) and small-molecule inhibitors that target cytokine pathways have been established as promising strategies in cancer therapy [93]. mAbs, such as those that neutralize IL-6 (e.g., tocilizumab) or TNF-α (e.g., infliximab), have demonstrated efficacy in reducing tumor-associated inflammation and improving outcomes in specific cancers. In contrast, small-molecule inhibitors frequently target intracellular signaling cascades downstream of cytokine receptors to facilitate oral bioavailability and broader tissue penetration [94]. IL-1 blockade, particularly targeting IL-1β, emerged as a potential anticancer strategy following the CANTOS trial, where canakinumab (a monoclonal anti-IL-1β antibody) reduced lung cancer incidence in patients with high-risk cardiovascular status [95]. This led Novartis to initiate a phase III trial (CANOPY-A) to evaluate canakinumab as adjuvant therapy for non-small cell lung cancer (NSCLC). However, in 2023, Novartis announced that the CANOPY-A trial failed to meet its primary endpoint, whereby it showed no significant improvement in disease-free survival as compared to placebo [96]. CANOPY-A was negative largely because a non-selected, adjuvant NSCLC population diluted any benefit of IL-1β blockade - an effect that seems restricted to inflammation-high, myeloid-driven biology and possibly more relevant to prevention/interception than to clearing micrometastases. Future success will hinge on biomarker-guided enrollment, biology-matched combinations, and choosing settings where IL-1 is clearly a driver. Thus, this outcome underscores the complexity of cytokine networks in cancer, where systemic IL-1 inhibition may not universally translate to therapeutic benefit despite initially promising findings. These results underscore the need for more precise biomarker-driven patient selection as well as combinatorial approaches in cytokine-targeted oncology.

## Conclusions

Cytokines play a pivotal role in the complex relationship between chronic inflammation and cancer. Although cytokines are essential for immune defense and tissue repair, their chronic activation can create a pro-tumorigenic environment that promotes cancer initiation and progression. Targeting cytokine signaling pathways represents a promising alternative strategy for treating inflammation-associated cancers, with several cytokine inhibitors already in clinical use or in clinical trials. As our understanding of the TME continues to evolve, therapeutic interventions that are aimed at modulating cytokine activity are likely to play an increasingly important role in cancer treatment.
